# Predominance of HBV Genotype B and HDV Genotype 1 in Vietnamese Patients with Chronic Hepatitis

**DOI:** 10.3390/v13020346

**Published:** 2021-02-22

**Authors:** Nghiem Xuan Hoan, Mirjam Hoechel, Alexandru Tomazatos, Chu Xuan Anh, Srinivas Reddy Pallerla, Le Thi Kieu Linh, Mai Thanh Binh, Bui Tien Sy, Nguyen Linh Toan, Heiner Wedemeyer, C.-Thomas Bock, Peter G. Kremsner, Christian G. Meyer, Le Huu Song, Thirumalaisamy P. Velavan

**Affiliations:** 1 Institute of Tropical Medicine, University of Tübingen, 72076 Tübingen, Germany; nghiemxuanhoan@gmail.com (N.X.H.); mirjam.hoechel@t-online.de (M.H.); alex_tomazatos@yahoo.com (A.T.); srinivas-reddy.pallerla@uni-tuebingen.de (S.R.P.); lk.linh1509@gmail.com (L.T.K.L.); maibinhtieuhoa108@gmail.com (M.T.B.); peter.kremsner@uni-tuebingen.de (P.G.K.); christian.g.meyer@gmail.com (C.G.M.); 2 Vietnamese-German Center for Medical Research, VG-CARE, Hanoi, Vietnam; bsxuananh108@yahoo.com.vn (C.X.A.); tiensy2015@yahoo.com (B.T.S.); lehuusong@108-icid.com (L.H.S.); 3 Institute of Clinical Infectious Diseases, 108 Military Central Hospital, Hanoi, Vietnam; 4 Department of Pathophysiology, Vietnam Military Medical University, Hanoi, Vietnam; toannl@vmmu.edu.vn; 5 Department of Gastroenterology, Hepatology and Endocrinology, Hannover Medical School, 30623 Hannover, Germany; Wedemeyer.heiner@mh-hannover.de; 6 Department of Infectious Diseases, Robert Koch Institute, 13353 Berlin, Germany; BockC@rki.de; 7 Centre de Recherches Medicales de Lambarene, Lambaréné, Gabon

**Keywords:** hepatitis D virus, hepatitis B virus, genotypes, Vietnam, hepatocellular carcinoma

## Abstract

Hepatitis delta virus (HDV) coinfection will additionally aggravate the hepatitis B virus (HBV) burden in the coming decades, with an increase in HBV-related liver diseases. Between 2018 and 2019, a total of 205 HBV patients clinically characterized as chronic hepatitis B (CHB; *n* = 115), liver cirrhosis (LC; *n* = 21), and hepatocellular carcinoma (HCC; *n* = 69) were recruited. HBV surface antigen (HBsAg), antibodies against surface antigens (anti-HBs), and core antigens (anti-HBc) were determined by ELISA. The presence of hepatitis B viral DNA and hepatitis delta RNA was determined. Distinct HBV and HDV genotypes were phylogenetically reconstructed and vaccine escape mutations in the “a” determinant region of HBV were elucidated. All HBV patients were HbsAg positive, with 99% (*n* = 204) and 7% (*n* = 15) of them being positive for anti-HBc and anti-HBs, respectively. Anti-HBs positivity was higher among HCC (15%; *n* = 9) compared to CHB patients. The HBV-B genotype was predominant (65%; *n* = 134), followed by HBV-C (31%; *n* = 64), HBV-D, and HBV-G (3%; *n* = 7). HCC was observed frequently among young individuals with HBV-C genotypes. A low frequency (2%; *n* = 4) of vaccine escape mutations was observed. HBV-HDV coinfection was observed in 16% (*n* = 33) of patients with the predominant occurrence of the HDV-1 genotype. A significant association of genotypes with alanine aminotransferase (ALT) and aspartate aminotransferase (AST) enzyme levels was observed in HBV monoinfections. The prevalence of the HDV-1 genotype is high in Vietnam. No correlation was observed between HDV-HBV coinfections and disease progression when compared to HBV monoinfections.

## 1. Introduction

Although safe and effective vaccines against infections with the hepatitis B virus (HBV) are available, it is estimated that in 2015, approximately 257 million people were living with chronic hepatitis B worldwide, with 887,000 fatalities [1]. Around 5% of these chronically HBV-infected individuals are estimated to be coinfected with the hepatitis delta virus (HDV) [2].

HBV is a hepatotropic enveloped DNA virus with a partially double-stranded circular genome containing four overlapping open reading frames [3]. HDV is an RNA virus unrelated to any other known RNA virus, which propagates as a satellite virus of HBV and accentuates the complications of viral hepatitis. HDV has a circular single-stranded negative RNA genome encoding the HDV antigen (HDAg) [4]. HDV requires the hepatitis B surface antigen for active replication in hepatocytes, which could be a coinfection with HBV or superinfection in patients already infected with HBV [5,6]. HBV infection leads to different clinical outcomes, ranging from chronic hepatitis B infection (CHB) to liver cirrhosis (LC) and hepatocellular carcinoma (HCC) [7]. Chronic HBV and HDV coinfection is associated with an almost threefold higher risk of the development of LC [8], a threefold higher risk of LC progressing to HCC, and a twofold increased mortality rate in LC patients compared to HBV monoinfected patients [9].

Genotypes HBV-A to HBV-D are the most widely spread variants across the world, while HBV-E to HBV-J have a smaller geographic coverage, with HBV-I and HBV-J being confined to eastern and southeast Asia (Vietnam, Laos, Japan) [10,11]. HDV is presently classified into eight genotypes (HDV-1 to HDV-8), of which HDV-1 has the most widespread geographic coverage [12,13]. The severity of viral hepatitis as well as the patients’ response to antiviral IFN-α therapy have been linked to distinct HBV genotypes [14]. HBV-C was found to be associated with an increased risk of HCC in comparison to HBV-B, especially in older patients [15,16,17,18]. In cases of HBV-HDV coinfection, the levels of alanine aminotransferase (ALT) and aspartate aminotransferase (AST) were higher than in HBV monoinfections [19,20]. In addition, as thrombocytopenia is common in HBV-infected patients with cirrhosis, the platelet (PLT) count is another important parameter proposed as an indicator of HBV disease progression [21,22].

Hepatitis B is endemic in Vietnam, with an estimated 10–20% of the general population (7–14 million) living with CHB [23]. Despite the fact that the HBV vaccination was initiated in 2003, Vietnam’s national coverage of the HBV vaccination is lagging behind WHO targets [24,25], and the burden of HBV-related liver disease is expected to rise [26]. Hepatitis B vaccination birth dose coverage increased to 75% in 2012 from 65% in 2006. Rapid genetic drift complemented by recombination events leads to the fixation of escape mutations (VEM) in the virus population, further challenging prevention [27,28]. The HBV genotypes HBV-B and HBV-C are the most widespread variants in Vietnam, of which HBV-B is dominant [29,30]. HDV-1 and HDV-2 are common genotypes in the north and the south of Vietnam, respectively. Reports from the last two decades show that the HDV prevalence in Vietnam varies from low (1.3%) [31,32] to recent high levels (10–15%) [19,33,34,35].

Given the endemicity of HBV in Vietnam and the assumed high prevalence of HDV, the objectives of this study were to determine the distribution of HBV and HDV genotypes in HBV-infected patients from northern Vietnam, and to determine the impact of individual genotypes and HBV-HDV coinfection on the progression of HBV-related liver disease. In addition, we sought to identify HBV S gene escape mutants that may render vaccines less effective or allow reactivation of hepatitis B.

## 2. Materials and Methods

### 2.1. Ethics Statement

The study was approved by the institutional review board of the 108 Military Central Hospital, Hanoi, Vietnam (108MCH/RES/Epi HBV-HDV-HEV D2-14-03-2014, 15 May 2015). Informed consent was obtained from all participants after detailed explanation of the study at the time of sampling.

### 2.2. Study Cohort

**Figure 1:**
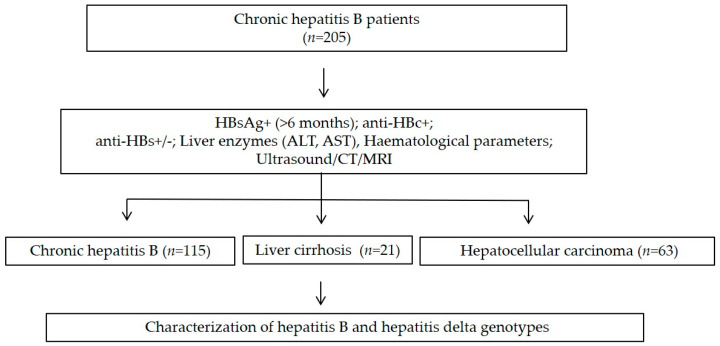
Flow chart of the study design.

A total of 205 HBV-positive patients from North Vietnam were recruited between 2018 and 2019 at the 108 Military Central Hospital in Hanoi, Vietnam. The demographic characteristics and medical history of the patients were collected. The patients were tested for serum HBsAg, ALT, AST liver function tests including PLT levels. Chronic HBV infection was diagnosed by the presence of HBsAg (>6 months) with anti-HBc IgG positive titers. Other laboratory assessments, including hematological, biochemical, molecular, and histological tests and imaging modalities were performed to establish the definitive diagnosis. The chronic HBV carriers were classified into three distinct groups based on clinical manifestations. Individuals with clinical symptoms of hepatitis (intermittently or persistently elevated liver enzymes (ALT, AST) were defined as chronic hepatitis B (CHB) patients. Patients with liver cirrhosis (LC) were diagnosed based on liver biopsy or imaging features of liver cirrhosis on ultrasound, and/or computed tomography (CT), and/or magnetic resonance imaging (MRI) in combination with abnormal liver function tests, portal hypertension with esophageal varices, splenomegaly and ascites. The hepatocellular carcinoma patients (HCC) were diagnosed according to the American Association for the Study of Liver Diseases (AASLD) guideline for HCC (Figure 1). Patients were divided into the clinical subgroups of chronic hepatitis B (CHB, *n* = 115), liver cirrhosis (LC, *n* = 21), and hepatocellular carcinoma (HCC, *n* = 69). All LC and HCC patients and most CHB patients were hospitalized for treatment. Blood samples were collected from all patients at admission and serum was separated and stored at −80 °C until further use.

### 2.3. Serological Assays

Anti-HBc and anti-HBs were detected using the Monolisa Anti-HBc PLUS and the Monolisa Anti-HBs PLUS kits (Bio-Rad, Feldkirchen, Germany), respectively. Optical density was measured on a CLARIOstar reader (BMG Labtech, Ortenberg, Germany). Positivity for anti-HBs was defined as an anti-HBs level ≥ 10 mIU/mL. HBsAg was detected by HBsAg one version ULTRA ELISA (DIA.PRO Milano, Italy), which has a diagnostic specificity of 99.5% and analytical sensitivity < 0.1 WHO IU/mL. This was performed following the manufacturer’s instructions.

### 2.4. Nucleic Acids Isolation

Total DNA and RNA was isolated from 200 μL serum using the QIAamp DNA and QIAamp Viral RNA MiniKits (Qiagen GmbH, Hilden, Germany), respectively, following the manufacturer’s instructions. Elution of DNA and RNA was done in a 50 μL elution buffer provided in the kits.

### 2.5. HBV and HDV Genotyping

Viral DNA was detected by an HBV-specific nested PCR assay as previously described [36], with minor modifications. The protocol targeted a 332 bp fragment of the HBV S gene, spanning the interval of nt 455 to 786 based on the HB057 reference strain (Genbank accession number HM011485). PCR amplification was carried out in a 25 μL reaction volume (5 µL DNA template, 0.2 mM dNTPs, 0.4 mM MgCl_2_, 0.3 µM of specific primers, 1x PCR buffer, 0.2 U Taq DNA polymerase (Qiagen GmbH, Hilden, Germany)). The first PCR assay was performed using the primers HBV-22, HBV-65, and HBV-66, and the second PCR assay was performed using the primers HBV-24, HBV-41, and HBV-64 (Table S1). Thermal cycling was initiated with denaturation for 15 min at 94 °C, followed by 35 cycles of denaturation for 30 s at 94 °C, annealing for 30 s at 55 °C (54 °C in the second PCR), and extension for 30 s at 72 °C, concluded with a final extension for 10 min at 72 °C (5 min in the second PCR). Both PCR rounds were performed in a Mastercycler Nexus (Eppendorf AG, Hamburg, Germany) with positive and negative controls.

HDV RNA was detected by amplifying a segment of the gene encoding the HDAg and its adjacent non-coding region. The amplicon was 235 bp long, spanning the interval of nt 888 to 1122 relative to the GenBank reference genome of NC_001653.2. Reactions were performed by a slightly modified HDV-specific nested OneStep RT-PCR assay as described previously [33]. The RT-PCR mixture was prepared using the QIAGEN OneStep RT-PCR Kit (Qiagen GmbH, Hilden, Germany) in a reaction volume of 12.5 µL (4 µL RNA template, 0.4 mM dNTPs, 0.6 µM HDV-04 and HDV-05 primers, 1x RT-PCR buffer plus enzyme mix). Thermal cycling parameters were as follows: reverse transcription for 30 min at 50 °C, initial denaturation for 15 min at 95 °C, followed by 40 cycles of denaturation for 10 s at 94 °C, annealing for 20 s at 61 °C, and extension for 30 s at 72 °C, terminated with a final extension for 2 min at 72 °C. The nested PCR was carried out in 10 µL reaction volume (1 µL template, 0.375 mM dNTPs, 0.25 mM MgCl_2_, 0.3 µM specific primers, 1x PCR buffer, Taq DNA polymerase (Qiagen GmbH, Hilden, Germany)) using HDV-06 and HDV-07 primers. Thermal cycling started with initial denaturation for 5 min at 95 °C, followed by 35 cycles of denaturation for 30 s at 94 °C, annealing for 30 s at 58 °C, and extension for 45 s at 72 °C, concluded with a final extension for 10 min at 72 °C.

PCR amplicons were visualized on 1.5% TBE agarose gels, purified with ExoSAP-IT PCR clean-up (ThermoFischer Scientific, Waltham, MA, USA), and sequenced on an Applied Biosystems 3130xl Genetic Analyzer (ThermoFischer Scientific, Waltham, MA, USA).

### 2.6. Sequence Data Analysis

The HBV and HDV sequences were analyzed and trimmed with Geneious Prime^®^ v2020.1.2 (Biomatters, Auckland, New Zealand). HBV and HDV genotypes were identified from sequences using the HBVdb online database [37] and the NCBI Blastn tool, respectively. Following genotyping, sequences were aligned with HBV and HDV sequences retrieved from NCBI GenBank. Maximum likelihood phylogenetic trees were inferred with PhyML 3.0 [38], using the K80 model of evolution [39]. Statistical support of tree nodes was assessed by approximated likelihood ratio tests (aLRT) and dendrograms were visualized with FigTree v1.4.4 [40]. Sequences in the present study have been submitted to GenBank and assigned the accession numbers MW044953-045136 for HBV and MW045137-045166 for HDV.

### 2.7. Statistical Analysis

Non-parametric data of quantitative variables between two or more than two groups were compared by Mann–Whitney-U or Kruskal–Wallis tests. Chi-square or Fisher’s exact tests were used to examine differences of categorical variables between two or more groups. Statistical significance was considered when *p* < 0.05. All statistical tests were computed with IBM SPSS Statistics v26 (IBM Corporation, Armonk, NY, USA).

## 3. Results

### 3.1. Baseline Characteristics of HBV Patients

**Figure 2:**
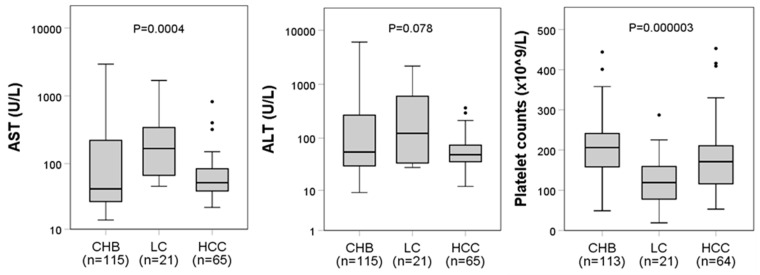
Association of enzyme and platelet levels with clinical diagnoses. Boxplots illustrate medians with 25 and 75 percentiles. (∙) *p*-values were calculated using the Kruskal–Wallis statistical test. Boxplots were created with SPSS (IBM Corp.). AST, aspartate amino transferase; ALT, alanine amino transferase; PLT, platelets.

Of the 205 HBV-infected patients aged 18–82 years with an average age of 49 years, the majority were males (77%, *n* = 158) (Table 1). As expected, patients with HCC were significantly older than those with LC (*p* = 0.007) or CHB (*p* < 0.0001). Serological assays showed that all patients were HBsAg-positive for six months. Fifteen patients (7%) were positive for anti-HBs and 204 (99%) were positive for anti-HBc. The AST levels and PLT counts differed significantly between the patient subgroups (*p* = 0.0004 and *p* < 0.0001, respectively), but no statistical difference was found for ALT levels (Figure 2). For patients with LC, post hoc analysis revealed that AST levels were higher compared to those of CHB patients (*p* < 0.0001). Furthermore, LC patients had lower PLT counts than HCC (*p* = 0.0047) and CHB patients (*p* < 0.0001). In individuals with HBV-HDV coinfection, we found no association of their infection status with liver enzyme levels or PLT counts (Table S2, Figure S1).

### 3.2. HBV and HDV Genotypes and Their Associations with the Outcomes of the Patients

**Figure 3:**
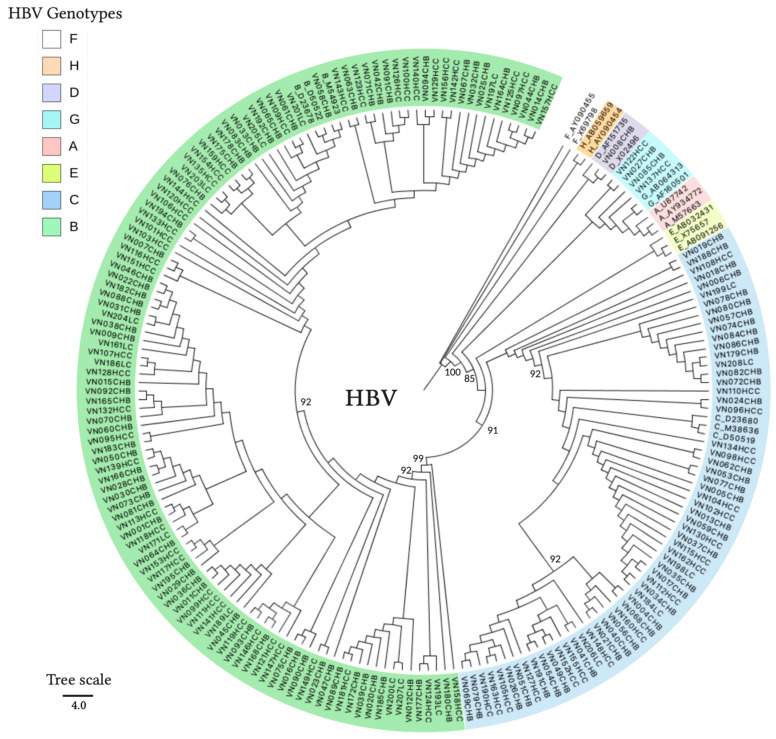
Maximum likelihood phylogenetic tree of HBV S-gene fragment (309 nucleotides). The reference sequences were retrieved from GenBank and labelled with the HBV genotypes and accession numbers. Generated sequences were labelled with the country code, sample ID, and diagnosis (e.g., VN001CHB = Vietnam 001 chronic hepatitis B). Node values indicate statistical support by approximate likelihood ratio test (aLRT) in PhyML 3.0. The scale bar indicates nucleotide substitutions per site.

**Figure 4:**
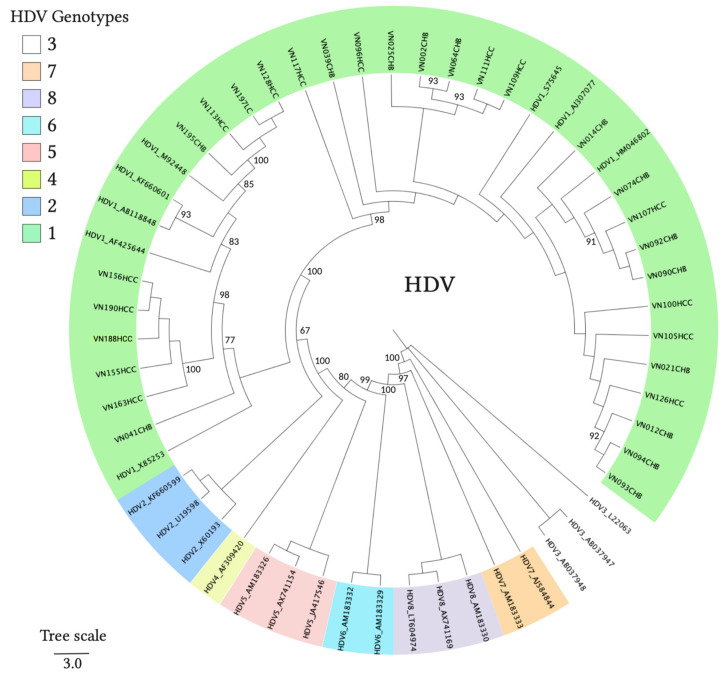
Maximum likelihood phylogenetic tree constructed using a fragment of the HDV genome (194 nucleotides). The reference sequences were retrieved from GenBank and labelled with the hepatitis delta virus (HDV) genotypes and the GenBank accession numbers. Sample sequences are labelled with the country code for Vietnam, the sample ID and the diagnosis. Node values indicate statistical support by approximate likelihood ratio test (aLRT) in PhyML 3.0. The scale bar indicates nucleotide substitutions per site.

The majority of patients were infected with the HBV-B genotype (65%, 134/205), followed by those infected with HBV-C (31%, 64/205), HBV-G (2%, 5/205), and HBV-D (1%, 2/205). HDV coinfection in HBV patients was detected in 16% (33/205) of the HBV patients and 30 positive samples were successfully genotyped as HDV-1; the remaining three samples yielded low-quality sequences which could not be reliably genotyped. Phylogenetic clustering of representative HBV and HDV sequences confirmed the genotype identity as assigned by database searches, indicating the presence of four HBV genotypes (HBV-B, -C, -D, and -G) and HDV genotype HDV-1 in our study group (Figure 3 and Figure 4).

The HBV-B genotype dominated in all three clinical groups (LC = 76%, HCC = 68%, CHB = 62%). HBV-C was present mostly in the CHB group (34%), followed by the HCC (29%) and LC groups (24%) (Table 1). However, we did not find significant differences regarding the presence of these major HBV genotypes in any of the patient groups (*p* = 0.37 for HBV-B, *p* = 0.58 for HBV-C). The majority of the HBV-B-positive patients had CHB without progression to LC or HCC (53%, 71/134). Over a third of the patients had HCC (35%, 47/134) and a smaller proportion had been diagnosed with LC (12%, 16/134). Of the 64 patients infected with HBV-C, 61% of patients (39/64) were diagnosed with CHB, 31% (20/64) with HCC, and 8% (5/64) with LC. The two patients infected with HBV-D had CHB. The few HBV-G infections were seen in the CHB (60%, 3/5) and the HCC (40%, 2/5) groups.

AST levels were higher in patients infected with HBV-B compared to HBV-C-positive patients (*p* = 0.044). Moreover, in the CHB group, the ALT levels were higher in HBV-B-infected individuals than in patients with HBV-C (*p* = 0.027). The PLT counts, however, showed no significant difference between the clinical groups.

The extent of HBV-HDV coinfections differed across the subgroups. The HCC group had the highest rate of HDV positivity (24%, 16/69). In the CHB patients, 14% (16/115) of patients had HDV coinfection, followed by the LC patients (5%, 1/21). When compared across different HBV genotypes, the coinfection rate of HDV-1 was similar for the two main HBV genotypes, with 16% (21/134) for HBV-B and 15% (10/65) for HBV-C. Finally, coinfection with HDV was found for one of each of the infrequent HBV genotypes, respectively (HBV-D 50%, 1/2 and HBV-G 20%, 1/5).

### 3.3. Hepatitis B Surface Antigen Escape Mutations

**Figure 5:**
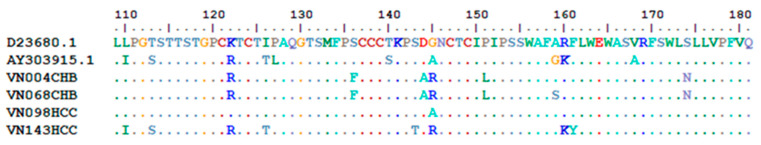
Alignment of the HBV sequences containing vaccine escape mutations. AY303915.1 represents an HBV vaccine escape mutant containing the G145A substitution [42]. Sites of vaccine escape mutations (aa144 and aa145) are highlighted in red.

In order to identify mutations in the HBV “a” determinant region that are associated with the HBV vaccine escape [41], sequenced fragments were translated in silico to amino acid sequences. Comparative analyses of HBV amino acid sequences revealed three residue changes commonly associated with vaccine escape in four HBV patients. The G145R, D144A, and G145A vaccine mutations were detected in 2% (4/205) of samples (Figure 5). None of the four patients were positive for anti-HBs.

## 4. Discussion

Despite a universal prevention program lasting almost two decades, Vietnam’s population is expected to be increasingly burdened by HBV-related illness by the mid-2020s [26]. The remarkably complex interaction of HBV with its host results in a multifaceted pathology that is often aggravated by coinfection with HIV and other agents causing viral hepatitis (HDV, HCV, HEV). Vertical transmission of HBV is of particular concern, as it is an important route of HBV transmission in Vietnam [43]. Hence, the latent character of CHB could sustain a rise in the incidence of LC and HCC in the coming years, alongside a decrease in HBV prevalence through vaccination.

We found a very high positivity rate for anti-HBc (99%). While the observation of HBsAg with or without anti-HBc indicates replication of the virus, the co-occurrence of anti-HBs and anti-HBc is considered a marker of HBV replication. Although the prevalence of anti-HBs (7%) was not significantly different across the three subgroups, it was significantly higher in patients with HCC compared to the CHB group, suggesting that the presence of anti-HBs may be indicative of HCC development. This concurs with the report of Jin et al., [44] who found that coexistence of low HBsAg levels and high anti-HBs levels might increase the risk of HCC development. Furthermore, the coexistence of HBsAg and anti-HBs was associated with an increased “a” determinant variability [45]. Of the 15 anti-HBs positive samples, 11 HBV sequences were successfully translated into protein sequences, but none showed a common vaccine escape mutation in the “a” determinant of the S gene, nor any substantial genetic variation.

HBV-B is known to be the most widespread HBV genotype in Vietnam, with detection rates >70% being reported repeatedly in the last decade [29,30,34,46]. In this context, the prevalence of HBV-B documented by the present study (65%) remains in the same order of magnitude and confirms the intense circulation of this genotype. To some extent, the same is true for the prevalence of HBV-C (31%), which is similar to that reported recently by Bui et al. [29] (27%), but considerably higher than in other recent studies (12–18%) [30,34,46]. Although HBV-D (1%) and HBV-G (2%) are not endemic in Vietnam, their detection is not completely unexpected, considering the extent of global transportation networks and the contemporary dynamics of infections at both regional and global scales.

Previous research found that HBV-C is associated with increased severity of liver disease [15], and a higher risk of developing HCC compared to that provided by HBV-B [18]. A study of 2762 HBV-positive Taiwanese patients found that the rate of HCC development was approximately 2.5 times higher for patients infected with HBV-C, compared to patients infected with genotype B [18]. Our investigation found higher enzyme levels in HBV-B infected patients compared to HBV-C-positive individuals, indicating an increased severity of liver damage. The frequency of HCC was higher in HBV-B than HBV-C infections, although this diagnosis was not correlated with the presence of a certain genotype.

HBV-B was previously shown to be associated with HCC development in patients <50 years of age, while the same progression was observed in patients >50 years with HBV-C infection [15,16]. Even with a higher frequency of HBV-C in younger HCC patients in our cohort, there was no significant difference in the genotype distribution within the HCC group, according to this age criterion. Nevertheless, the age of HBV-C-infected HCC patients (average 54 years) was significantly lower than the age of HCC patients infected with HBV-B (average 61 years), indicating that linkage of HBV-B with early-onset HCC is not generally valid. We hypothesize that HBV seroprevalence may be low in the younger age group (<18 years) and therefore fewer HDV infections occur because an HBV vaccination program has been implemented in Vietnam since 2003.

As expected in an HBV-endemic region, the common vaccine escape mutations D144A, G145R, and G145A were detected in the “a” determinant region of the HBV S gene from two, three, and one of 184 analyzed sequences, respectively. None of the four HBV patients showing these escape mutations were positive for anti-HBs.

HDV-1 and HDV-2 are the most common HDV genotypes in Vietnam, having a clear, distinct geographic distribution. HDV-2 is found mostly in southern parts of the country [35] and HDV-1 is circulating predominantly in the north, where Binh et al. [33] found a remarkably high HDV seroprevalence in Hanoi (30%). Our results confirm this spatial pattern, as well as the high level of virus activity in the population [19,33,34,35]. The marked difference between the HDV prevalence recorded in the last decades [31,32] may reflect the inclusion of high-risk cohorts from urban centers [34]. Moreover, HDV coinfection often results in a decrease in HBV viral loads and higher rates of antigen negativity compared to HBV monoinfections, potentially precluding detection of the HBV infection [34]. HDV was most frequently detected in the HCC patients, at a rate of almost double that observed in the CHB group. Although our results concur with investigations conducted in Europe by showing an increased HCC risk in HBV-HDV coinfection [9], previous studies from Vietnam have yielded conflicting results [19,33]. Therefore, further studies are required for a clearer conclusion regarding the relationship of HCC and LC development in HBV-HDV coinfections.

With regard to HBV genotypes, HDV showed an almost identical distribution between HBV-B positive patients (15%) and those infected by HBV-C (16%); as a result of the small number of HBV-D and HBV-G positives, their relation to HDV infection remains, however, unclear. Despite earlier reports of increased disease severity in HBV-HDV-coinfections relative to HBV monoinfections [47,48] and elevated levels of AST and ALT in Vietnamese and Chinese cohorts [19,20], we did not observe significant differences between the enzyme levels of both monoinfected and coinfected patients. Our results, however, are in accordance with recent investigations from Vietnam which found no correlation between the infection status and enzymatic activity [33]. In the same Vietnamese cohort, PLT counts were shown to be significantly lower in HBV-HDV coinfected patients [33]. Even if the mean PLT count in our study cohort was slightly lower in the coinfected group, the difference was not significant. A factor that may account for the association’s significance is that the cohort analyzed by Binh et al. contained a high proportion of LC patients, which is generally the clinical group with the lowest PLT counts.

In summary, the distribution of HBV genotypes found in this study is consistent with other studies from Vietnam. We did not find specific associations between HBV genotypes and disease severity, in particular with HCC development. Our results do not confirm a role of HBV-C in progression towards HCC in older patients, as in this cohort the HCC patients infected with HBV-B were significantly older than the HBV-C-positive patients. Commonly reported VEMs are present in the HBV population circulating in Vietnam. Upon screening for HDV RNA, we found a relatively high virus prevalence, confirming HDV-1 as the main genotype in northern Vietnam. However, no correlation was found between HDV-HBV coinfection and increased severity of liver disease when compared to HBV monoinfection. Considering HDV activity remains at high levels in Vietnam, our results warrant the implementation of wide-scale testing for a clearer assessment of HDV coinfection rates, along with stepping up the effort to improve HBV vaccine coverage, especially in infants.

## Figures and Tables

**Table 1:** Clinical characteristics of 205 hepatitis B virus (HBV)-infected patients.

Characteristics/Parameters	Total (*n* = 205)	CHB (*n* = 115)	LC (*n* = 21)	HCC (*n* = 69)	*p*-Value
**Age (years)**	49 [36–61]	40 [31–53]	45 [39–59]	61 [53–67]	<0.0001 *
**Male/female**	158/47	78/37	17/4	63/6	0.0011 ^#^
**AST (U/L)**	54 [32–157]	42 [27–221]	167 [67–342]	52 [39–84]	0.0004 *
**ALT (U/L)**	55 [34–157]	55 [30–269]	123 [34–601]	49 [36–74]	0.0778 *
**PLT (×10^9^/L)**	187 [142–229]	206 [158–241]	119 [78–159]	171 [118–210]	<0.0001 *
**Anti-HBc (+/−)**	204/1	115/0	20/1	69/0	N/A
**Anti-HBs (+/−)**	15/190	5/110	1/20	9/60	0.0743 ^~^
**HBV genotypes (B/C/D/G)**	134/64/2/5	71/39/2/3	16/5/0/0	47/20/0/2	N/A
**HDV coinfections**	33	16	1	16	N/A

CHB, chronic hepatitis B; HCC, hepatocellular carcinoma; LC, liver cirrhosis; AST, aspartate amino transferase; ALT, alanine amino transferase; PLT, platelets. Values given are medians and interquartile ranges. * Kruskal–Wallis test; ^#^ Chi-square test; ^~^ Fisher’s exact test.

## Data Availability

The data supporting reported results are available on request.

## Supplementary Materials

The following are available online at https://www.mdpi.com/1999-4915/13/2/346/s1, Supplemental Methods description. Figure S1: Association of enzyme and platelet levels with coinfection. Table S1: Primer sequences for HBV and HDV genotyping. Table S2: Clinical characteristics of HBV monoinfected and HBV-HDV coinfected patients.
