# Supplementary material for: Predominance of HBV Genotype B and HDV Genotype 1 in Vietnamese Patients with Chronic Hepatitis
Source: Viruses. 2021 Feb 22;13(2):346. doi: 10.3390/v13020346 (PMC7926858; doi:10.3390/v13020346)
Supplement: Supplementary file 1 [file viruses-13-00346-s001.pdf]

**Supplementary Materials**

**Tables**

**Table S1.** Primer sequences for HBV and HDV genotyping.

| Primer | Sequence (5′-3′) | Position | PCR round |
|---|---|---|---|
| HBV-22_F | TGCTGCTATGCCTCATCTTC | 414-433 | 1st round PCR |
| HBV-24_F | CAAGGTATGTTGCCCGTTTGTCCT | 455-478 | 2nd round PCR |
| HBV-41_R | GGACTCACGATGCTGTACAG | 767-787 | 2nd round PCR |
| HBV-64_R | GGACTCAMGATGYTGCACAG | 767-787 | 2nd round PCR |
| HBV-65_R | CAAAGACAAAAGAAAATTGG | 803-822 | 1st round PCR |
| HBV-66_R | CACAGATAACAAAAAATTGG | 803-822 | 1st round PCR |
| HDV-04_F | GGATGCCCAGGTCGGACCG | 856–874 | 1st round PCR |
| HDV-05_R | AAGAAGAGTAGCCGGCCCGC | 1159–1179 | 1st round PCR |
| HDV-06_F | ATGCCATGCCGACCCGAAGA | 888–907 | 2nd round PCR |
| HDV-07_R | GGGGAGCGCCCGGDGGCGG | 1104–1122 | 2nd round PCR |

Positions are given according to HBV genome HM011485 and HDV genome LC_001653. F: forward primer, R: reverse primer.

**Table S2.** Clinical characteristics of HBV monoinfected and HBV-HDV coinfected patients.

| Characteristics | Monoinfection (n=172) | Coinfection (n=33) | *P*-value |
|---|---|---|---|
| Age (years) | 49 [36-61] | 48 [40-62] | 0.587ß |
| Male/Female | 130/42 | 28/5 | 0.246# |
| AST (U/L) | 55 [32-167] | 44 [35-82] | 0.396ß |
| ALT (U/L) | 53 [34-169] | 57 [39-78] | 0.897ß |
| PLT (x$10^9$/L) | 191 [142-229] | 178 [141-220] | 0.630ß |
| Anti-HBc (+/-) | 171/1 | 33/0 | N/A |
| Anti-HBs (-/+) | 160/12 | 30/3 | N/A |

AST: aspartate amino transferase; ALT: alanine amino transferase; PLT: platelets. Values given are medians and interquartile ranges. ß Mann–Whitney-U-Test; # Chi-square test.

**Figure**

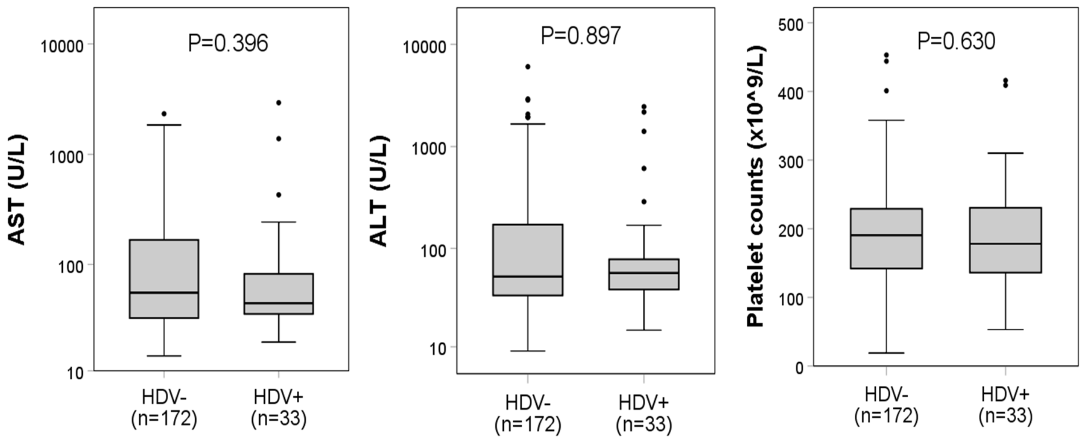


**Figure S1. Association of enzyme and platelet levels with coinfection.** Boxplots illustrate medians with 25 and 75 percentiles. *P*-values were calculated using the Kruskal–Wallis test. Boxplots were created with SPSS (IBM Corp.). AST, aspartate amino transferase; ALT, alanine amino transferase; PLT, platelets.
